# Estimation of Solar Radiation for Tomato Water Requirement Calculation in Chinese-Style Solar Greenhouses Based on Least Mean Squares Filter

**DOI:** 10.3390/s20010155

**Published:** 2019-12-25

**Authors:** Dapeng Zhang, Tieyan Zhang, Jianwei Ji, Zhouping Sun, Yonggang Wang, Yitong Sun, Qingji Li

**Affiliations:** 1 College of Information and Electrical Engineering, Shenyang Agricultural University, Shenyang 110866, China; nyebo@syau.edu.cn (D.Z.); jjw@syau.edu.cn (J.J.); 2012500039@syau.edu.cn (Y.W.); 2017188004@stu.syau.edu.cn (Y.S.); 2 School of Automation and Electrical Engineering, Shenyang Ligong University, Shenyang 110159, China; 3 College of Horticulture, Shenyang Agricultural University, Shenyang 110866, China; sunzp@syau.edu.cn; 4 National and Local Joint Engineering Research Center of Northern Horticultural Facilities Design and Application Technology (Liaoning), Shenyang 110866, China; 5 Key Laboratory of Protected Horticulture, Ministry of Education, Shenyang 110866, China

**Keywords:** CSG, tomato water requirement calculation, LMS filter, solar radiation estimation

## Abstract

The area covered by Chinese-style solar greenhouses (CSGs) has been increasing rapidly. However, only a few pyranometers, which are fundamental for solar radiation sensing, have been installed inside CSGs. The lack of solar radiation sensing will bring negative effects in greenhouse cultivation such as over irrigation or under irrigation, and unnecessary power consumption. We aim to provide accurate and low-cost solar radiation estimation methods that are urgently needed. In this paper, a method of estimation of solar radiation inside CSGs based on a least mean squares (LMS) filter is proposed. The water required for tomato growth was also calculated based on the estimated solar radiation. Then, we compared the accuracy of this method to methods based on knowledge of astronomy and geometry for both solar radiation estimation and tomato water requirement. The results showed that the fitting function of estimation data based on the LMS filter and data collected from sensors inside the greenhouse was y = 0.7634x + 50.58, with the evaluation parameters of R^2^ = 0.8384, rRMSE = 23.1%, RMSE = 37.6 Wm^−2^, and MAE = 25.4 Wm^−2^. The fitting function of the water requirement calculated according to the proposed method and data collected from sensors inside the greenhouse was y = 0.8550x + 99.10 with the evaluation parameters of R^2^ = 0.9123, rRMSE = 8.8%, RMSE = 40.4 mL plant^−1^, and MAE = 31.5 mL plant^−1^. The results also indicate that this method is more effective. Additionally, its accuracy decreases as cloud cover increases. The performance is due to the LMS filter’s low pass characteristic that smooth the fluctuations. Furthermore, the LMS filter can be easily implemented on low cost processors. Therefore, the adoption of the proposed method is useful to improve the solar radiation sensing in CSGs with more accuracy and less expense.

## 1. Introduction

Since being introduced in the 1930s, the Chinese-style solar greenhouse (CSG) has gradually grown in Chinese agriculture. The greenhouse vegetable industry accounts for 20% of the total vegetable production area in China, but it produces 35% of the output and 60% of the economic value in 2013 [1]. In 2013, the CSG cultivation area amounted to 612,000 ha [2].

Solar radiation is an important factor affecting the calculations of water requirement [3,4] and environmental evaluation [5] in precision agriculture. However, few CSGs are equipped with enough sensors due to purchase price and subsequent maintenance [6,7]. Solar radiation sensors, among the sensors in solar greenhouse applications, are very expensive [8,9,10,11]. To produce quality crops in a sufficient quantity in greenhouses, the demand for solar radiation sensing in CSGs is increasing rapidly [12,13,14]. To address this need, some methods that estimate the solar radiation inside CSGs (H_i_) with few or no sensors have been introduced. Ahamed et al. (2018) proposes the CSGHEAT model in their study and this model can estimate H_i_ based on outdoor solar radiation and cloud cover [15]. Tong et al. (2009), Ahamed (2018) et al. use outdoor solar radiation and film transmittance to estimate H_i_ [16,17]. In addition, some researches about indoor solar radiation estimation on other type greenhouses are also performed [18,19].

In studies of Tong (2009), Ahamed et al. (2018) and Sethi (2009), the horizontal solar radiation outside the greenhouse (H_out_) is decomposed into beam radiation (H_b_) and diffuse radiation (H_d_) [15,16,18]. In the study of Gavilán (2015), decomposition is not conducted [19].

For decomposition, H_out_ is first divided into H_b_ and H_d_ [16,17,20]. Inman et al. (2013) proved that the ratio of the clearness index (K_t_) of H_out_ to extraterrestrial solar radiation (H_o_) is an important factor for decomposition [21]. According to K_t_, the ratio of H_d_ to H_out_ is a fixed function relationship [22,23]. After decomposition, H_b_ and H_d_ are multiplied by film transmittance of beam radiation (τ_b_) and film transmittance of diffuse radiation (τ_d_), respectively. Finally, the sum of the two multiplications is the estimation of radiation inside a greenhouse. In our study, the estimation of H_i_ estimated by astronomy and geometry method is presented as H_c_. In the other case, H_out_ is directly multiplied by greenhouse transmittance (τ_g_) so decomposition is not needed [19]. In summary, the accuracy of these methods is related to K_t_, τ_b_, τ_d,_ or τ_g_, and τ_b_, τ_d_, or τ_g_ are always constants. However, none of these parameters are constant in practice, reducing the accuracy of these methods [24,25,26].

At the experimental site, the low outside temperature in winter means ventilation is rare, so properly calculating the water requirement for the tomatoes is important because overestimating the requirement will lead to higher humidity, which is harmful to tomatoes [27].

Some models of crop water requirement have been formulated, such as the Penman–Monteith equation [28,29] and the Hargreaves equation [30], which are based on meteorological parameters including solar radiation, temperature, relative humidity, wind speed, etc. However, the Penman–Monteith equation was proven to be restricted because too many sensors, which are costly and require frequent maintenance, are needed in greenhouse applications [31]. Hargreaves and Allen (2003) proved the accuracy of the Hargreaves equation is related with the calculation period, which must be five days or more, so it is not suitable for high-frequency irrigation applications [32]. In many cases, tomatoes are cultivated in substrates with lower water-holding capacity, so high-frequency irrigation is required.

To address the restrictions of the Penman–Monteith and Hargreaves equations in greenhouse applications, an equation was proposed by Carmassi et al. (2007) [33]. To perform Carmassi’s equation, only solar radiation and temperature are needed.

To the best of our knowledge, adaptive filters have rarely been applied in solar radiation estimation. Among digital filters used in applications, the adaptive filter [34,35] is advantageous compared with the finite impulse response (FIR) filter and infinite impulse response (IIR) filter due to its better performance in situations with a spectrum overlap between the signal and noise [36]. Adaptive filters are widely used in many fields such as noise canceling, system identification, and signal prediction [37,38].

Therefore, in our study, we adopted an adaptive filter to estimate H_i_ more accurately and less expensively. The objectives of this study were (1) to estimate H_i_ and compare the results conducting estimations with other methods (2) to calculate the water required by tomatoes using the H_i_ estimated with an adaptive filter and compare the results with the requirement calculated using other methods and (3) to analyze the performance of the proposed method.

## 2. Material and Methods

### 2.1. Experimental Materials, Measurement and Evaluation

#### 2.1.1. CSG Architecture

**Figure 1:**
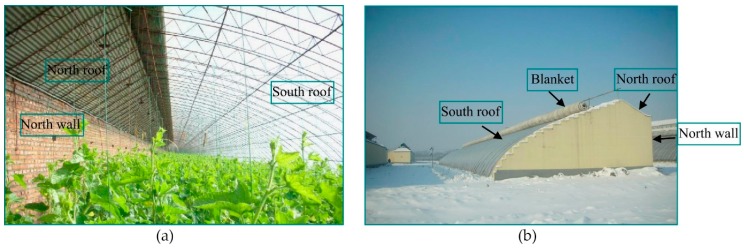
Pictures of the (**a**) inside and (**b**) outside of a Chinese-style solar greenhouse (CSG) [6].

As shown in Figure 1, the architecture of a CSG consists of a south roof, north roof, north wall, gables, and blanket. In many cases, a CSG is east-west oriented to intercept more solar energy. In the cold season, the blanket is rolled up during the day and the sunlight enters from the south roof. The crops, ground, north wall, and gables absorb energy. After the blanket is dropped at night, the gables and north wall release heat to maintain temperature [39].

CSGs extend the crop growing season in the cold areas in China between 34° and 41° N, where the temperature falls below −20 °C at night. CSG cultivation requires little auxiliary heating equipment; the consumption of energy and emissions of carbon dioxide are considerably reduced.

#### 2.1.2. Experimental Site and Measurement Methods

We conducted this study in an east-west-oriented CSG in Shenyang, China (41°48′ N, 123°24′ E, 42 m a.s.l.). The greenhouse was 60 m long and 12 m wide. The height of the north wall and north roof were 3 m and 5.5 m, respectively. The south roof was covered by a single layer of 0.00012 m thick polyethylene film.

The cultivation area inside the greenhouse was 55 m long from east to west and 10 m wide. Tomatoes were grown with row spacing of 1 m, within-row spacing of 0.33 m, and plant density of 4 plants·m^−2^. Tomatoes were grown in substrate and irrigated using a drip irrigation system. The tomatoes were sown on 5 August 2017. Then, the transplant was performed on 1 September 2017 and the cultivation finished on 27 December 2017. We conducted this study using H_out_ data collected from outer weather station and indoor pyranometers (H_s_) from 1–26 December 2017. It was cold and nature ventilation was rare during the experimental days.

Six temperature sensors (SHT10, Sensirion, Zurich, Switzerland) and three pyranometers (MP200, Apogee Instruments, Logan, UT, USA) were installed in the experimental greenhouse. The temperature sensors were hung 1.5 m above the ground and pyranometers were placed horizontally at different heights above the ground (1.5, 2, and 2.5 m) according to the growth condition of the tomatoes. The placement of indoor sensors is shown in Figure S1.

The temperature sample interval was 15 min and the mean of the temperature recorded by the six installed sensors was considered the temperature inside the greenhouse. The sample interval of solar radiation was 15 min [40] and the mean of three installed pyranometers was taken as the true H_i_ value.

#### 2.1.3. Evaluation Parameters

As evaluation parameters, we adopted the coefficient of determination (R^2^), percent error (PE), root mean square error (RMSE), relative root mean square error (rRMSE), and mean absolute error (MAE), and these parameters were calculated according to Equations (1)–(5), respectively [22,23,40]. In our study, R^2^, RMSE, rRMSE, and MAE were firstly used for comparisons between the estimated solar radiation and measured solar radiation. Additionally, they were also used to compare the daily water requirements of tomatoes calculated by estimated data and sensor data. RE was used to compare the error rates of water requirements.

(1) $$R^{2}=1-\frac{\sum {\left(y_{e}-y_{m}\right)}^{2}}{\sum \left(y_{m}-y_{m\_\mathrm{mean}}\right)}$$

(2) $$\mathrm{Percent}\;\mathrm{Error}\left(\mathrm{PE}\right)=\frac{y_{e}-y_{m}}{y_{m}}\times 100\%$$

(3) $$\mathrm{RMSE}=\sqrt{\frac{\sum {\left(y_{e}-y_{m}\right)}^{2}}{n}}$$

(4) $$\mathrm{rRMSE}=\frac{100}{y_{m\_\mathrm{mean}}}\sqrt{\frac{\sum {\left(y_{e}-y_{m}\right)}^{2}}{n}}$$

(5) $$\mathrm{MAE}=\frac{\sum \left|y_{e}-y_{m}\right|}{n}$$

where y_e_ is the estimated value, y_m_ is measured value, y_m_mean_ is the mean of y_m_, and n is the number of samples.

### 2.2. Classic Methods of Estimating H_i_

Two methods have mainly been used for estimating H_i_. Method 1 is based on knowledge of astronomy and geometry according to the following procedure:

Step 1: Calculate H_0_ using Equation (6) [12]:

(6) $$H_{0}=\frac{24\times 3600}{\pi }G_{\mathrm{sc}}\left(1+0.033\cos\frac{360n_{\mathrm{day}}}{365}\right)\times \left(\cos\varphi \cos\delta \sin\omega_{s}+\frac{\pi \omega_{s}}{180}\sin\varphi \sin\delta \right)$$

where G_sc_ is solar constant, G_sc_ = 1367 Wm^−2^, ϕ is the latitude of the location and n_day_ is the day number of the year, counted from 1 January, and δ and ω_s_ are the daily solar declination and sunset hour angle, respectively [22]:

(7) $$\delta =23.45\sin\left[\frac{360\left(n_{\mathrm{day}}+284\right)}{365}\right]$$

(8) $$\omega_{s}=\cos^{-1}\left(-\tan\varphi \tan\delta \right)$$

Step 2: Calculate K_t_ according to H_o_ and H_out_ via Equation (9) [22]:

(9) $$K_{t}=\frac{H_{\mathrm{out}}}{H_{0}}$$

Step 3: Decompose H_out_ into H_b_ and H_d_ according to K_t_ [8,13] using Equation (10):

(10) $$\frac{H_{d}}{H_{\mathrm{out}}}=\{\begin{array}{cc} 0.95 & K_{t}<0.175\\ 0.9698+0.4353K_{t}-3.4499K_{t}^{2}+2.1888K_{t}^{3} & 0.175<K_{t}<0.775\\ 0.26 & K_{t}>0.775. \end{array}$$

Step 4: Calculate H_c_ via Equation (11) [15,17]:

(11) $$H_{c}=H_{c}\tau_{b}+H_{d}\tau_{d}=\left(H_{\mathrm{out}}-H_{d}\right)\tau_{b}+H_{d}\tau_{d}$$

where τ_b_ is film transmittance to H_b_ and τ_d_ is film transmittance to H_d_. The values of τ_b_ and τ_d_ are 0.88 and 0.65 in the experimental greenhouse, respectively.

Method 2 [19] is based on Equation (12):

(12) $$H_{c}=H_{\mathrm{out}}\tau_{g}$$

Method 2 is simpler than Method 1, but as shown in Figure S2, the mean and variance τ_g_ of the experimental greenhouse was different from that in another study [19], so Method 1 was adopted in this study for comparison.

In both methods above, film transmittance and global transmittance are constant, but this does not reflect the reality. Some studies proved global transmittance changed with the incidence angle of the sun [24,25] and film transmittance changed due to aging and deposition [26]. So, estimating solar radiation merely according to fixed transmittance is not reliable and is prone to error.

### 2.3. Tomato Water Requirement Calculation

Carmassi’s equation was dedicated to calculating the water requirement of tomatoes according to only two meteorological parameters: solar radiation and temperature. Carmassi’s equation is calculated as follows:

Step 1: Calculate leaf area index (LAI) according to Equation (13) [33]:

(13) $$\{\begin{array}{c} \mathrm{LAI}=-a+\frac{b+a}{1+\exp[\left(c-\mathrm{GDD}\right)/d]}\\ \mathrm{GDD}=\sum_{\mathrm{Start}\_\mathrm{day}}^{\mathrm{Stop}\_\mathrm{day}}\left(T_{\mathrm{avg}}-8\right) \end{array}$$

where a, b, c, and d are regression constants, a = 0.335, b = 4.803, c = 755.3, and d = 134.7; GDD is tomato growing degree days; T_avg_ is indoor daily average temperature, °C; Start_day presents the sowing date and Stop_day presents the date when cultivation ends. Something to be pointed out is that GDD is taken as dimensionless in LAI’s computation. The GDD values are shown in Figure S3, and the values of GDD on 1 and 26 December were 1252 and 1340, respectively.

Step 2: Calculate extinction factor k according to Equation (14) [33]:

(14) $$\frac{H_{\mathrm{up}}}{H_{\mathrm{down}}}=\exp\left(-k\times \mathrm{LAI}\right)$$

where H_up_ and H_down_ are solar radiation above and below the canopy, respectively; H_up_ and H_down_ were measured using two pyranometers placed horizontally at 2.5 and 1.5 m above the ground. During the experimental days, k was 0.69.

Step 3: Calculate the water requirement of tomatoes according to Equation (15) [33]:

(15) $$V_{u}=A\left[1-\exp\left(-k\times \mathrm{LAI}\right)\right]\frac{R_{i}}{\lambda^{*}}$$

where A = 0.946, B = 0.188, λ* is the latent heat of vaporization (2.45 MJ kg^−1^), and R_i_ is the energy intercepted by canopy (MJ m^−2^ day^−1^). R_i_ is calculated as [33]:

(16) $$R_{i}=\sum_{T_{\mathrm{start}}}^{T_{\mathrm{stop}}}H_{i}T_{s}$$

where T_s_ is the sample interval, and T_start_ and T_stop_ are start time and stop time of water requirement calculation, respectively. T_start_ was confirmed by the time when H_s_ first rose above 10 Wm^−2^ and T_stop_ was confirmed by the time when the H_s_ first fell to 0 Wm^−2^. On sunny days during the experiment, T_start_ was 08:00 and T_stop_ was 16:00.

### 2.4. LMS Filter

According to the filter refresh algorithm, some kinds of adaptive filters can be used [41], such as the least mean squares (LMS) filter [35], recursive least squares (RLS) filter [42], least mean p-norm (LMP) filter [43], normalized LMP (NLMP) filter [44], least mean absolute deviation (LMAD) filter [44], and normalized LMAD (NLMAD) filter [44]. The basic diagram of adaptive filter is shown in Figure S4. Among these, the LMS filter’s resource consumption is low, making it suitable for applications in resource-constrained systems such as microcontrollers, which have only smaller RAM and run at a lower speed [45]. So, we only focused on the LMS filter’s performance.

The LMS filter updates its filter coefficients according to least mean squares algorithm; computation proceeds according to Equations (17)–(19) [36]:

(17) $$y\left(n\right)=x^{T}\left(n\right)w\left(n\right)$$

(18) e(n)=x(n)−d(n)

(19) w(n+1)=w(n)+2μe(n)x(n)

where μ is the convergence factor of LMS filter, and w(n + 1) is the filter coefficient in the next iteration.

μ, which is related to convergence speed and approximate precision, is an important factor in the LMS filter. In addition, a smaller value of μ leads to higher approximate precision and lower convergence speed, and vice versa. The LMS filter is fully analyzed according to Equations (20)–(22) [37,38]:

(20) $$E\left[w\left(n+1\right)\right]=E\left[w\left(n\right)\right]+2\mu E\left[e\left(n\right)x\left(n\right)\right]=E\left[w\left(n\right)\right]+2\mu R_{\mathrm{dx}}-2\mu R_{\mathrm{xx}}E\left[w\left(n\right)\right]$$

where R_dx_ = E[d(n)x(n)] is the cross correlation matrix of the input and desired signals, R_xx_ = E[x(n)x^T^(n)] is the autocorrelation matrix of the input signal, and R_xx_ is at least a positive semi-definite matrix, so a normalized orthogonal matrix Q sets up Equation (21) [37,38]:

(21) $$R_{\mathrm{xx}}=Q\Lambda Q^{-1}=Q\Lambda Q^{T}$$

where the modal matrix Q is orthonormal. The columns of Q, which are the eigenvectors of R_xx_, are mutually orthogonal and normalized. Notice that Q^−1^ = Q^T^, Λ is the spectral matrix and all its elements are zero except for the main diagonal, whose elements are the set of eigenvalues of R_xx_, which are presented as, λ_1_, λ_2_, λ_3_, …, λ_L_. According to Equation (22), Λ has the following form [37,38]:

(22) $$\Lambda =\left[\begin{array}{ccccccc} \lambda_{1} & & 0 & & 0 & & 0\\ & & & \cdots & & & \\ 0 & & \lambda_{2} & & 0 & & 0\\ & \vdots & & \ddots & & \vdots & \\ 0 & & 0 & & \lambda_{L-1} & & 0\\ & & & \cdots & & & \\ 0 & & 0 & & 0 & & \lambda_{L} \end{array}\right]$$

The eigenvalues of R_xx_ are all real and greater or equal to zero, and μ can be calculated according to [37,38]:

(23) $$0<\mu <\frac{1}{\lambda_{\max}}$$

where λ_max_ is the maximum eigenvalue of R_xx_.

A tightly-constrained equation about μ is [37,38]:

(24) $$0<\mu <\frac{1}{\mathrm{tr}(R_{\mathrm{xx}})}$$

where tr(R_xx_) is the trace of R_xx_. And tr(R_xx_) is calculated as [37,38]:

(25) $$\mathrm{tr}\left(R_{\mathrm{xx}}\right)=LR_{\mathrm{xx}}\left(0\right)=LE\left[x^{2}\left(n\right)\right]$$

where L is the number of taps of the LMS filter.

Equations (24) and (25) prove that the upper bound of μ is the power of the input signal, which can be calculated easily in applications.

### 2.5. Discrete Fourier Transform (DFT), Fast Fourier Transform (FFT), and Pass Band Characteristics of Filters

#### 2.5.1. DFT and FFT

DFT is a fundamental tool in signal processing applications, and FFT [46] is the fast algorithm of DFT. The characteristics in the frequency domain of the interested signals are obtained by DFT and FFT. DFT is calculated according to [46]:

(26) $$X\left(k\right)=\sum_{n=0}^{N-1}x\left(n\right)e^{-j\frac{2\pi }{N}\mathrm{nk}}$$

where X(k) is the frequency domain values of time series x(n), N is the calculation length, and both n and k range from 0 to N − 1.

#### 2.5.2. Filter Pass Band Characteristic

The pass band characteristic of a filter is the response effect of the amplitude-frequency characteristic of a filter to the signal of a different frequency. It can be calculated using Equation (26). Filters are classified as high pass, low pass, band pass, notch, and all pass according to the band characteristics. For example, a low pass filter passes low-frequency components and attenuates high-frequency components, so the output signal is always smoother than the input signal.

### 2.6. Proposal Methods and Evaluation Procedures

We focused on estimating H_i_ using H_out_ recorded from a weather station, which is basic equipment in many growing areas in China.

**Figure 2:**
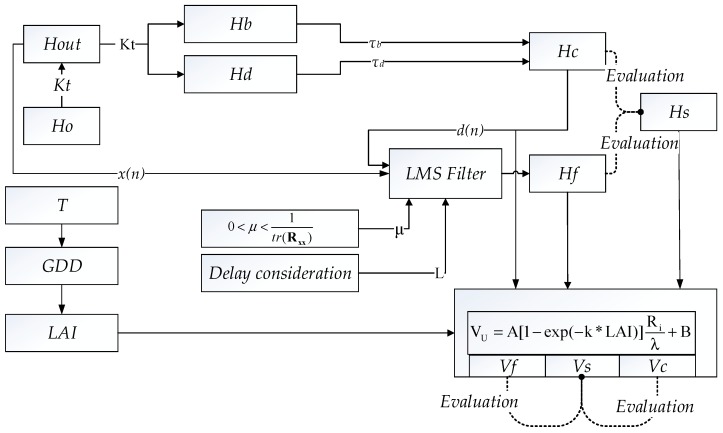
Flow chart of solar radiation estimation, water requirement calculation, and corresponding evaluations in this study. LMS = least mean squares.

The flow chart of data processing is shown in Figure 2. Firstly, H_out_ and H_s_ were obtained from a weather station and the sensors inside the greenhouse. H_c_ was calculated according Equations (6)–(11). Secondly, H_out_ and H_c_ were used as the x(n) and d(n) input signals for the LMS filter, respectively; therefore, the output signal of the LMS filter was the estimation of H_i_ and is presented as H_f_. The required water volume for tomato, according to H_c_, H_f_, and H_s_, which are presented as V_c_, V_f_, and V_s_, respectively, were calculated via Equations (13)–(16).

The performance of curve fitting, including H_f_–H_s_ and H_c_–H_s_, were evaluated to analyze each solar radiation estimation method. The performance of curve fitting, including V_f_–V_s_. and V_c_–V_s_, were evaluated to analyze water requirement according to each solar radiation estimation method. Both of the evaluations were based on the equations proposed in Section 2.1.3. All data in this study were processed and all figures were drawn using Python 3.7 (Python Software Foundation, Wilmington, DE, USA). The data of H_s_ and H_out_ is available in Data file S1 and Data file S2 respectively. And the program is available in Program file S1. 

## 3. Results and Discussion

### 3.1. Determination of μ and L

The length (L) of the LMS filter varies in different applications and ordinary lengths are 8, 9, 64, and 128, but the sample interval in greenhouse applications always ranges from 1 min to 1 h or more. Given a sample interval of 15 min, L = 128, introduces a time delay at more than 24 h, so a smaller number of taps is preferred. In this study, L was 9.

According to Equations (24) and (25), the upper bound of μ was computed. In experimental days, the maximum of E[x^2^(n)] was 145. To avoid computational overflow in the program, each value of H_out_ was multiplied by 0.01. So, the upper bound of μ was 0.0007.

In the range of 10^−5^ to 5 × 10^−4^, six values were chosen for evaluation to determine the exact value of μ. According to the procedure proposed in Section 2.6, H_f_ and H_c_ were computed according to H_out_ collected between 07:00 and 17:00 on experimental days. Then, the performance was evaluated; notably, in the three taps of the left shift of H_f_ for compensation of computational delay. In the latter parts of this study, the length and direction of shift were constant unless otherwise mentioned.

As shown in Table 1, the distribution of evaluation parameters showed a single peak, and R^2^, RMSE, rRMSE, and MAE reached their minimum when μ was 5 × 10^−5^; so, in this study, μ was determined.

### 3.2. Estimation of H_i_ and Tomato Water Requirement Calculation under Sunny, Partly Cloudy and Overcast Conditions

#### 3.2.1. Estimation of H_i_

**Figure 3:**
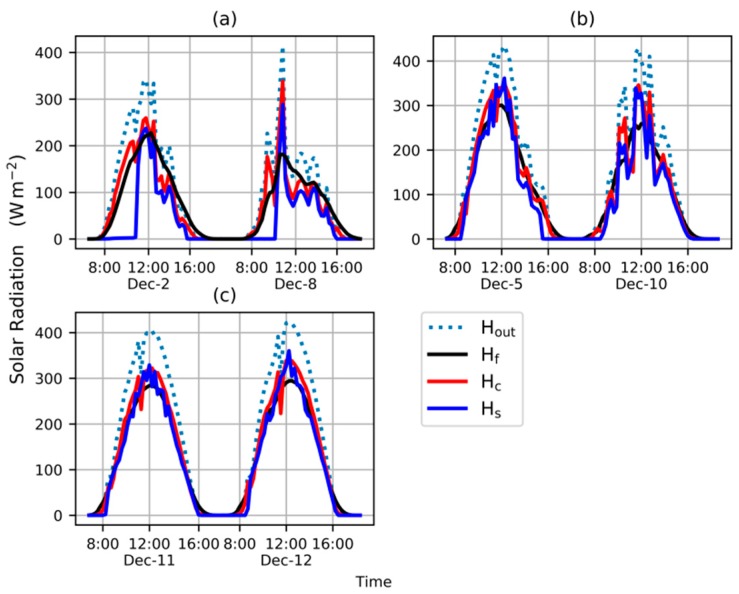
Solar radiation under different weather conditions (**a**) overcast (**b**) partly cloudy (**c**) sunny.

Computations were performed using data from six days according to the procedure in Section 2.6. The curves of H_out_, H_c_, H_f_, and H_s_ are shown in Figure 3. Two days were sunny, 11 and 12 December. In Figure 3c, the curve of H_s_ fluctuated obviously near its peak value, whereas the curve of H_f_, which tended to have a half-wave sinusoidal shape, was smooth. In other words, the fluctuation range of H_f_ was very narrow near noon. The fluctuations of H_out_ and H_c_ ranged between H_s_ and H_f_. We observed quick changes in the curves of H_out_ and H_c_ near their peak value at about 11:00 each day and the quick changes were introduced by a metal bar installed nearby the weather station. So, the weather station measurements were temporarily disturbed by the shadow of the bar. In contrast, the curve of H_f_ proved to be immune to this temporary disturbance.

The weather was partly cloudy on 5 and 10 December. According to the curves of H_out_, H_c_, H_f_, and H_s_ in Figure 3b, as the cloud cover increased after 14:00 on 5 December, the curves of H_out_, H_c_, and H_s_ fluctuated considerably, and these fluctuations made the curves rougher than the H_f_ curve. The overall trend of the curves was H_f_ > H_c_ > H_s_ during this time. The fluctuations of the curves of H_out_, H_c_, and H_s_ on 10 December were more obvious than on 5 December. However, the H_f_ curve was smoother than the other curves and fluctuation range was narrow on 10 December. In addition, the shape of H_f_ on both days distorted gradually.

The day was overcast on 2 and 8 December. Due to the lower outer solar radiation in the morning on these days, the blanket was rolled up later than usual. So a distinguishing rising edge, after which H_s_ curve was close to the H_c_ and H_f_ curves, rapidly appeared on the H_s_ curve in Figure 3a. The operation of the blanket resulted in a T_start_ at 11:00 and 10:00 on 2 and 8 December, respectively. According to the H_out_, H_c_, H_f_, and H_s_ curves in Figure 3c, as the cloud cover increased after 13:00 on 2 December, the curves of H_out_, H_c_, and H_s_ fluctuated obviously, making the curves rougher than the H_f_ curve. The fluctuations of H_out_, H_c_, and H_s_ curves on 8 December proved to be more obvious than on 2 December. However, the curve of H_f_ was smoother than other curves and the fluctuation range was narrow on 8 December. The shapes of H_f_ on both days were distorted and were no longer half-wave sinusoidal.

#### 3.2.2. Tomato Water Requirement

According to Equation (16), R_i_ was computed using H_c_, H_f_, and H_s_, which are presented as R_c_, R_f_, and R_s_, respectively. T_start_ values were 08:15, 08:00, 08:00, 08:00, 11:00, 10:00 and T_stop_ values were 16:00, 16:15, 15:45, 16:15, 15:45, 16:00 on 11, 12, 5, 10, 2, 8 December, respectively. Then, V_c_, V_f_, and V_s_. were calculated using Equation (15) as shown in Table 2. The PE of V_f_–V_s_. and V_c_–V_s_, presented as PE_fs_ and PE_cs_, respectively, calculated via Equations (27) and (28), respectively, are also shown in Table 2.

(27) $${\mathrm{PE}}_{\mathrm{fs}}=\frac{V_{f}-V_{s}}{V_{s}}\times 100\%$$

(28) $${\mathrm{PE}}_{\mathrm{cs}}=\frac{V_{c}-V_{s}}{V_{s}}\times 100\%.$$

The data on 11 and 12 December in Table 2 show that V_s_. < V_f_ < V_c_ and PE_fs_ < PE_cs_. The PE_fs_ values on both days were smaller than 2% but the values of PE_cs_ tended to be more than 7%. The data show PE_fs_ < PE_cs_ on 5 December and PE_fs_ > PE_cs_ on 10 December. The difference in PE_fs_ and PE_cs_ was 5.6% on 5 December and 1.2% on 10 December. The data on 2 and 8 December show that PE_fs_ = PE_cs_.

### 3.3. Overall Performance of Estimation of H_i_ and Tomato Water Requirement Calculation

#### 3.3.1. Overall Performance of Estimation of H_i_

**Figure 4:**
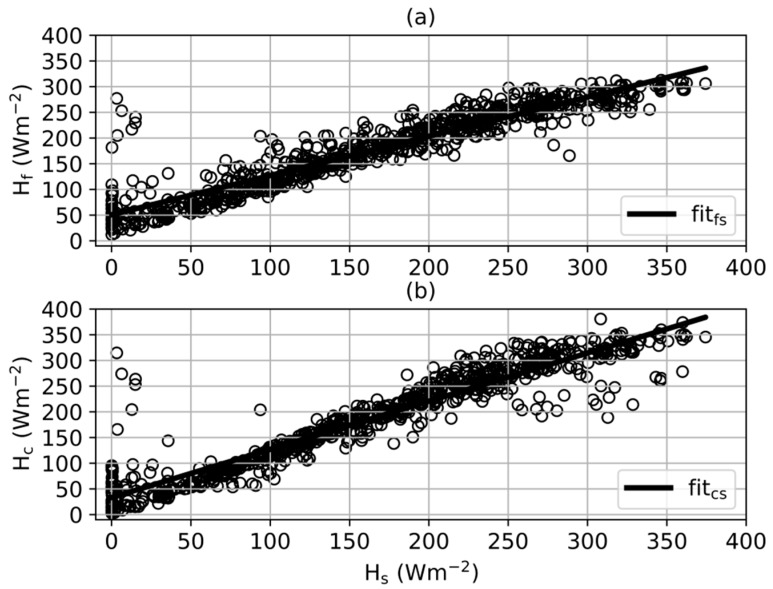
Scatter plots of (**a**) H_s_ vs. H_f_ and (**b**) H_c_ vs. H_f_, fitted are also shown in each sub-figure.

The scatter plot of H_f_ and H_s_ is shown in Figure 4a and the fitting function of H_f_ and H_s_ was y = 0.7634x + 50.58. The scatter plot of H_c_ and H_f_ is shown in Figure 4b and the fitting function was y = 0.9376x + 33.04. The latter analysis focuses on the performance of each method under sunny conditions, which dominated during the experimental period.

In Figure 4a,b, when some points of H_f_ increased above 30 Wm^−2^, H_s_ was nearly 0 Wm^−2^. These larger values are mainly attributed to the postponed blanket operations. Differences of H_s_ and estimated values including H_f_ and H_c_ appeared to be large because H_f_ and H_c_ had reached higher values when H_s_ was still near 0 Wm^−2^.

As shown in Figure 4a, H_s_ increased to about 200 Wm^−2^ during 09:00–10:00 and 14:00–15:00 when H_s_ stayed below H_f_ and H_c_.

In Figure 4a, as H_s_ rose above 200 Wm^−2^, the number of points of H_f_ < H_s_ also increased gradually. When H_s_ rose above 300 Wm^−2^, the number of points of H_f_ < H_s_ was greater than the number of points of H_f_ > H_s_. The details of the curves during 10:00–14:00 on 11 and 12 December indicates as H_s_ rose above 200 Wm^−2^, the increasing speed rose so the curve of H_s_ gradually stayed above the curve of H_f_. Hence, the increasing number of points of H_f_ < H_s_ in Figure 3c contributed to the increasing rising speed of H_s_. The peaks in the H_s_ curve on these two days in Figure 4a reached more than 300 Wm^−2^; so, when H_s_ > 300 Wm^−2^, the number of points of H_f_ < H_s_ dominated. Oscillation was observed when H_s_ rose above 200 Wm^−2^, so some H_f_ > H_s_ points were introduced.

In contrast, in Figure 4b, the number of points of H_c_ < H_s_ changed within a small variation range, and H_s_ stayed above H_c_ only near its peak value. When H_s_ rose above 250 Wm^−2^ (Figure 4b), some points of H_c_ < H_s_ occurred due to the disturbance caused by the metal bar near the outer weather station.

In summary, the overall trend in Figure 4a was H_c_ > H_f_ > H_s_ when H_s_ < 200 Wm^−2^, and H_c_ > H_s_ > H_f_ when H_s_ > 200 Wm^−2^. According to Figure 4a,b, the fluctuation range of H_f_ was the smallest among the four curves.

**Figure 5:**
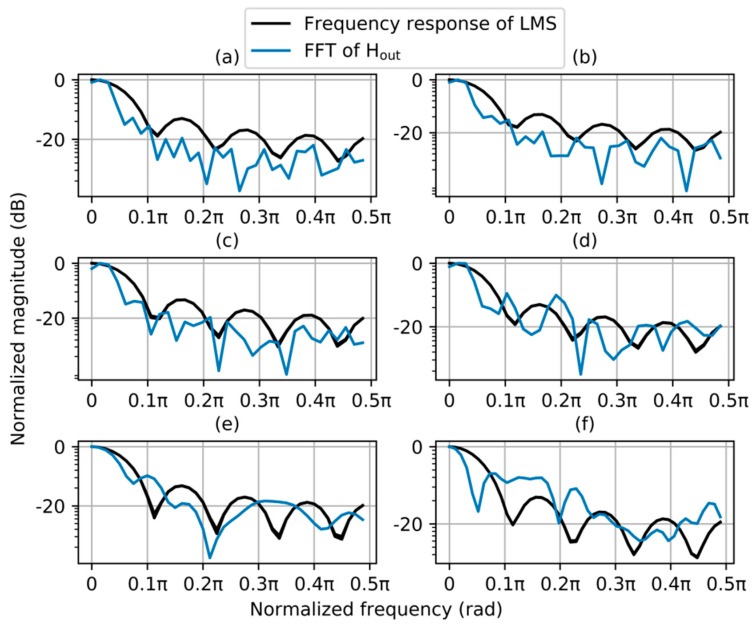
Frequency responses of LMS filter on sunny, partly cloudy, and overcast days, (**a**) 11, (**b**) 12, (**c**) 5, (**d**) 10, (**e**) 2, and (**f**) 8 December.

The pass band characteristics of LMS filters in Section 3.2 are shown in Figure 5. And the FFTs of H_out_ in Section 3.2 are also shown in this figure. The pass band characteristics of LMS filters in these six days were all low pass. The low pass characteristic made H_f_ smoother than H_out_, which was the smoothest among H_out_, H_c_, and H_s_. The low pass characteristic also made the LMS filter immune to temporary disturbances, which were common in many greenhouse applications.

We found the LMS filter is not applicable if research focuses on the fluctuations in solar radiation due to its low pass characteristic.

#### 3.3.2. Overall Performance of Tomato Water Requirement Calculation

**Figure 6:**
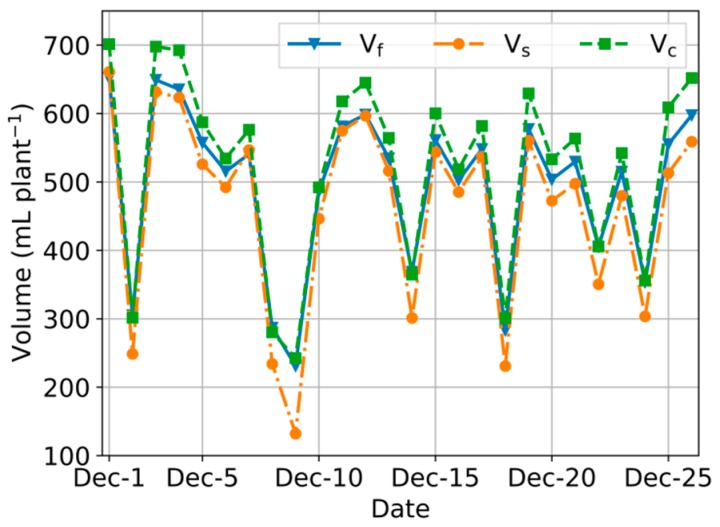
Tomato daily water requirements calculated according to different solar radiation estimation methods during experimental period, and water requirement computed according to data collected from sensors inside the greenhouse.

According to the procedure in Section 3.2.2, we calculated the V_c_, V_f_, and V_s_. of each day during the experimental period, as shown in Figure 6. The overall trend of these three values was the same: they all increased when cloud cover decreased. V_f_ was close to V_s_. when cloud cover was lower, and to V_c_ when cloud cover was higher.

**Figure 7:**
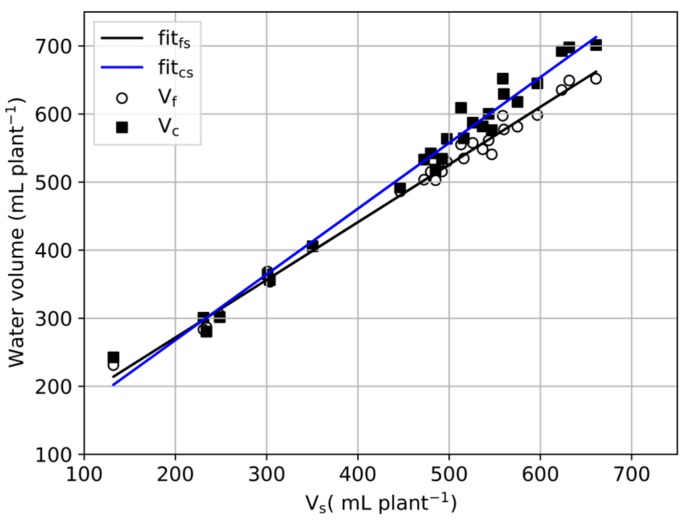
Scatter plot of V_f_–V_s_. and V_c_–V_s_. during experimental days.

The scatter plots of V_f_–V_s_. and V_c_–V_s_. are shown in Figure 7, and the fitting functions of V_f_–V_s_. and V_c_–V_s_. are y = 0.8470x + 102.2 and y = 0.9656x + 74.6, respectively. As shown in Figure 7, V_f_ was close to V_c_ when V_s_. < 400 mL and V_f_ was close to V_s_. when V_s_. > 400 mL. The difference between V_f_ and V_s_. decreased as V_s_. increased but, conversely, the trend in the difference between V_c_ and V_s_. was not the same as V_f_–V_s_. When V_s_. rose above 600 mL plant^−1^, the values of V_f_ and V_s_. were almost the same; when V_s_. dropped below 400 mL plant^−1^, the values of V_f_ and V_c_ were almost the same.

**Figure 8:**
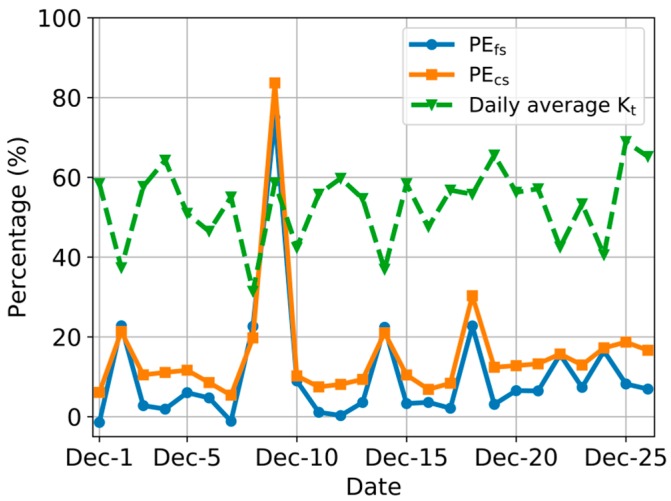
Daily average K_t_ and PE_fs_, PE_cs_ during experimental period.

PE_fs_, PE_cs_, and K_t_ are shown in Figure 8. We found an opposite trend between PEs and K_t_. The lowest value of PE_fs_ was lower than that of PE_cs_. Among 2, 8, 14, 22, and 24 December, in which K_t_ was greater than 40%, PE_fs_ was close to PE_cs_ and PE_fs_ < PE_cs_ on other days. Among 1, 2, 11, and 12 December, PE_fs_ was almost 0.

## 4. Discussions

The four evaluation parameters of H_f_–H_s_ and H_c_–H_s_ on days in Section 3.2 were computed according to Equations (1) and (3)–(5), as shown in Table 3. Studies proved that estimation of solar radiation in hourly intervals was good enough if rRMSE ranged from 34% to 41% [16,47,48], so conclusions can be drawn as follows. Under sunny conditions, the estimation of H_f_–H_s_ was more accurate than H_c_–H_s_ and both of the methods were good enough on 11 and 12 December. Under partly cloudy conditions, the estimations of H_f_–H_s_ and H_c_–H_s_ were all good enough, with rRMSE in both cases below 41% on 5 December. Due to the rRMSE of H_f_–H_s_ being above 41%, only the estimation of H_c_–H_s_ was good enough on 10 December. Under overcast conditions, the estimations of H_f_–H_s_ and H_c_–H_s_ were all poor, with rRMSE above 41% on 2 December and rRMSE of H_f_–H_s_ above 41%. Only estimation of H_c_–H_s_ was acceptable on 8 December.

Badescu et al. (2013, 2014) and Son et al. (2018) prove the estimation accuracy of solar radiation decreases with increasing cloud cover [47,48,49]. Additionally, the decreasing of estimation accuracy lies in the increasing portion of diffuse solar radiation, which is always measured at a lower accuracy [48]. Since outer solar radiation is decomposed into beam and diffuse parts in our study, estimation accuracy is also affected if cloud cover increases. However, with the LMS filter being introduced, estimation accuracy of ours is affected less than in other methods because of the LMS filter’s low pass characteristic. In contrast, the estimation accuracy of H_c_–H_s_ proved to be the best on partly cloudy days, the worst on sunny days, and medium on overcast days. In the study of Huang et al. (2019) and Tong et al. (2017) the fluctuation of H_i_ tends to be larger at noon [14,25], and the same trend was found in our study. It is a common phenomenon that large fluctuations appear in the data on a sunny noon, but to our knowledge the mechanism of this phenomenon needs further analysis.

The evaluation parameters of H_f_–H_s_, H_c_–H_s_, V_f_–V_s_. and V_c_–V_s_. during experimental days computed according to Equations (1) and (3)–(5) are shown in Table 4. The results indicate that estimation of H_f_–H_s_ was more accurate than H_c_–H_s_, so the proposed method proves to be more accurate than astronomy and geometry method [16,17]. Additionally, rRMSE of H_f_–H_s_ is within the range between 34% and 41% [16,48], so this method is acceptable in solar radiation estimation. In the study of Ahamed et al. (2018), the evaluation parameters of solar radiation estimation proved to be R^2^ = 0.71, RMSE = 68.34 Wm^−2^, and rRMSE = 30.54% in contrast [16]. Moreover, film transmittances are still important factors in our method and their values are not constant in applications, therefore, the accuracy of our model is affected by transmittances variations.

In the studies of Gueymard and Myers (2008), data filtering is considered as an important factor to improve solar radiation sensing accuracy [50]. In our study, the LMS filter performs low pass filtering and the operation of Equation (16) is also low pass filtering. So, the calculation of V_f_ performs a two-stage low pass filtering and the evaluation parameters of V_f_–V_s_. tend to be even better. The results of our study show the impacts introduced by fluctuations, especially in cloudy and overcast weather conditions. The results of our study have also proved the importance of filtering in both solar radiation estimation and water requirement calculation.

However, the following points need to be fully improved. Firstly, the accuracy of the model is affected by cloud cover. For better performance, the new methods in the measure/estimate diffuse of solar radiation with more accuracy are to be developed. Secondly, real-time transmittance computation should be an important part of the model for better accuracy. Thirdly, we conduct research supposing the CSG is at east-west orientation with no incline. However, some CSGs are built with an incline due to terrain restrictions. Finally, long term analysis is to be conducted for better use of our model.

In our study, with no indoor sensors needed, the cost of solar radiation sensing is little. In the literature of Villarrubia et al. (2017), the cost of an irrigation system is acceptable when cost amounts to 100€/250 m^2^ [51]. Therefore, our research reduces the expense of solar radiation sensing and is contributing to the promotion of precision agriculture in CSGs.

## 5. Conclusions

In this study, solar radiation inside a CSG was estimated based on an LMS filter, and then the tomato water requirement was calculated according to the estimation data. The performance of both solar radiation estimation and water requirement calculation were compared to the corresponding methods based on knowledge of astronomy and geometry.

The results showed that the fitting function of estimation data based on the LMS filter and data collected from sensors inside the greenhouse was y = 0.7634x + 50.58, with the evaluation parameters of R^2^ = 0.8384, rRMSE = 23.1%, RMSE = 37.6 Wm^−2^, and MAE = 25.4 Wm^−2^. The fitting function of the water requirement calculated according to the proposed method and data collected from sensors inside the greenhouse was y = 0.8550x + 99.10 with the evaluation parameters of R^2^ = 0.9123, rRMSE = 8.8%, RMSE = 40.4 mL plant^−1^, and MAE = 31.5 mL plant^−1^.

The low pass characteristic of the LMS filter leads to the following two results. First, the performance of the proposed method is more accurate than that of the contrastive method. Second, the proposed method performs well on sunny days but performs worse on party cloudy and overcast days. In addition, LMS is easy to be performed in microcontrollers. Therefore, the method is proved to be efficient and low cost in both solar radiation estimation and tomato water requirement calculation. However, it is not applicable if focusing on the fluctuations of solar radiation inside a greenhouse.

## Figures and Tables

**Table 1:** Evaluation parameters computed by different values of μ.

Evaluation Parameter	M					
	10^−5^	2 × 10^−5^	5 × 10^−5^	10^−4^	2 × 10^−4^	5 × 10^−4^
R^2^	0.3959	0.8031	0.8384	0.8254	0.7554	0.6010
RMSE (Wm^−2^)	72.7	41.5	37.6	39.1	46.2	59.0
rRMSE (%)	44.6	25.5	23.1	23.4	28.4	36.2
MAE (Wm^−2^)	58.7	30.2	25.3	25.8	33.6	45.9

**Table 2:** Evaluation parameters of tomato water requirements calculated according to different estimation methods of H_i_ under different conditions.

Date	Calculated Value				
	V_c_ (mL·Plant^−1^)	V_s_ (mL·Plant^−1^)	V_f_ (mL·Plant^−1^)	PE_fs_ (%)	PE_cs_ (%)
**2 December**	301.6	248.6	307.2	23.5	21.3
**8 December**	280.2	234.0	288.9	23.3	23.5
**5 December**	587.1	525.9	557.5	6.0	11.6
**10 December**	492.0	446.3	486.3	9.0	10.2
**11 December**	617.7	574.9	585.4	1.8	7.4
**12 December**	645.1	596.7	602.8	1.0	8.1

**Table 3:** Evaluation parameters of solar radiation estimation under different weather conditions.

Date		Evaluation Parameter			
		R^2^	RMSE (Wm^−2^)	rRMSE (%)	MAE (Wm^−2^)
**2 December**	H_f_–H_s_	0.4123	57.3	47.5	37.2
	H_c_–H_s_	0.6535	44.0	36.4	29.9
**8 December**	H_f_–H_s_	0.1153	55.5	59.6	38.6
	H_c_–H_s_	0.7767	27.9	30.0	11.8
**5 December**	H_f_–H_s_	0.9056	33.5	19.6	27.4
	H_c_–H_s_	0.8972	34.8	20.4	28.0
**10 December**	H_f_–H_s_	0.7909	43.9	31.5	34.4
	H_c_–H_s_	0.9393	23.7	16.7	18.5
**11 December**	H_f_–H_s_	0.9630	18.1	9.8	14.3
	H_c_–H_s_	0.6231	50.3	31.6	50.3
**12 December**	H_f_–H_s_	0.9525	21.1	10.7	15.5
	H_c_–H_s_	0.6147	51.0	30.6	51.0

**Table 4:** Evaluation parameters of solar radiation estimation and tomato water requirement calculation.

Evaluation Parameter	H_f_–H_s_	H_c_–H_s_	Evaluation Parameter	V_f_–V_s_	V_c_–V_s_
R^2^	0.8384	0.8084	R^2^	0.9123	0.7598
RMSE (Wm^−2^)	37.6	40.9	RMSE (mL·plant^−1^)	40.4	64.8
rRMSE (%)	23.1	25.1	rRMSE (%)	8.8	14.1
MAE (Wm^−2^)	25.4	29.6	MAE (mL·plant^−1^)	31.5	58.6

## Supplementary Materials

The following are available online at https://www.mdpi.com/1424-8220/20/1/155/s1, Figure S1: Diagram of sensor placement inside the greenhouse, Figure S2: Daily average greenhouse global transmittance (τg) calculated from data from exterior and interior inside sensors and the overall average value of τg during experiment, Figure S3: Growing degree days (GDD) during experimental days, Figure S4: Basic diagram of adaptive filter. Data file S1: Indoor solar radiation and temperature data file, values_in_copy.xlsx, Data file S2: Outside solar radiation data file, values_out_12.xlsx. Program file S1: LMS function and outcome display program file, lmsRev3.py.

## Nomenclature

H_s_	solar radiation inside greenhouse measured by sensors (Wm^−2^)
H_f_	solar radiation inside greenhouse estimated by LMS filter (Wm^−2^)
H_c_	solar radiation inside greenhouse estimated by astronomy and geometry method (Wm^−2^)
H_out_	horizontal solar radiation outside the greenhouse (Wm^−2^)
H_0_	extraterrestrial solar radiation (Wm^−2^)
K_t_	clearness index (dimensionless)
H_i_	solar radiation inside greenhouse (Wm^−2^)
H_b_	beam part of extraterrestrial solar radiation (Wm^−2^)
H_d_	diffuse part of extraterrestrial solar radiation (Wm^−2^)
τ_b_	film transmittance of beam radiation (dimensionless)
τ_d_	film transmittance of beam radiation (dimensionless)
τ_g_	greenhouse transmittance (dimensionless)
R_i_	energy intercepted by canopy (MJ m^−2^ day^−1^)
R_s_	energy intercepted by canopy calculated by sensor data (MJ m^−2^ day^−1^)
R_f_	energy intercepted by canopy calculated by LMS method (MJ m^−2^ day^−1^)
R_c_	energy intercepted by canopy calculated by astronomy and geometry method (MJ m^−2^ day^−1^)
V_u_	water requirement volume (mL plant^−1^)
V_s_	water requirement volume calculated by sensor data (mL plant^−1^)
V_f_	water requirement volume calculated by LMS method (mL plant^−1^)
V_c_	water requirement volume calculated by astronomy and geometry method (mL plant^−1^)
G_sc_	solar constant (1367 Wm^−2^)
δ	daily solar declination (degree)
ω_s_	sunset hour angle (degree)
ϕ	latitude of the location (degree)
n_day_	the day number of the year (dimensionless)
LAI	leaf area index (dimensionless)
k	extinction factor (dimensionless)
GDD	growing degree days (dimensionless)
T_s_	sampling interval(s)
T_start_	start time of water requirement calculation (hh: mm)
T_stop_	stop time of water requirement calculation (hh: mm)
μ	step size of LMS filter (dimensionless)
λ*	latent heat of vaporization (2.45 MJ kg^−1^)
L	length of LMS filter (dimensionless)
λ	eigenvalue of auto correlation matrix of the input signal
R_xx_	auto correlation matrix of the input signal
R_dx_	cross correlation matrix of the input and desired signals
PE_cs_	water volumes percent error calculated according to astronomy and geometry method and sensors data (dimensionless)
PE_fs_	water volumes percent error calculated according to LMS method and sensors data (dimensionless)

## References

[1] Liang L., Ridoutt B.G., Lal R., Wang D., Wu W., Peng P., Zhao G. (2019). Nitrogen footprint and nitrogen use efficiency of greenhouse tomato production in North China. J. Clean. Prod..

[2] Wang T., Wu G., Chen J., Cui P., Chen Z., Yan Y., Zhang Y., Li M., Niu D., Li B. (2017). Integration of solar technology to modern greenhouse in China: Current status, challenges and prospect. Renew. Sustain. Energy Rev..

[3] Gocic M., Motamedi S., Shamshirband S., Petkovic D., Chintalapati S., Hashim R., Arif M. (2015). Soft computing approaches for forecasting reference evapotranspiration. Comput. Electron. Agric..

[4] Petkovic D., Gocic M., Trajkovic S., Shamshirband S., Motamedi S., Hashim R., Bonakdari H. (2015). Determination of the most influential weather parameters on reference evapotranspiration by adaptive neuro-fuzzy methodology. Comput. Electron. Agric..

[5] Zhang X., Lv J., Dawuda M.M., Xie J., Yu J., Gan Y., Li J. (2019). Innovative passive heat-storage walls improve thermal performance and energy efficiency in Chinese solar greenhouses for non-arable lands. Sol. Energy.

[6] Tong G., Christopher D.M., Li T., Wang T. (2013). Passive solar energy utilization: A review of cross-section building parameter selection for Chinese solar greenhouses. Renew. Sustain. Energy Rev..

[7] Bontsema J., Van Henten E.J., Gieling T.H., Swinkels G.L. (2011). The effect of sensor errors on production and energy consumption in greenhouse horticulture. Comput. Electron. Agric..

[8] Ma J., Bi Z., Shi Y., Man K.L., Pan X., Wang J. OL-SVR based soft-sensor for real-time estimation of solar irradiance. Proceedings of the 2016 IEEE Asia Pacific Conference on Circuits and Systems (APCCAS).

[9] Lopezlapena O., Pallasareny R. (2018). Solar energy radiation measurement with a low-power solar energy harvester. Comput. Electron. Agric..

[10] Kipp and Zonen. http://www.kippzonen.com/ProductGroup/3/Pyranometers.

[11] Pieters J., Deltour J.M. (1999). Modelling solar energy input in greenhouses. Sol. Energy.

[12] Sonsteby A., Solhaug K.A., Heide O.M. (2016). Functional growth analysis of ‘Sonata’ strawberry plants grown under controlled temperature and daylength conditions. Sci. Hortic. Amst..

[13] Zhong P., Yang S., Qiao R., Wang T. (2011). Effect of light intensity on main quality of strawberry. Southwest China J. Agric. Sci..

[14] Huang S., Yan H., Zhang C., Wang G., Acquah S.J., Yu J., Opoku Darko R. (2020). Modeling evapotranspiration for cucumber plants based on the Shuttleworth-Wallace model in a Venlo-type greenhouse. Agric. Water Manag..

[15] Ahamed M.S., Guo H., Tanino K.K. (2018). Development of a thermal model for simulation of supplemental heating requirements in Chinese-style solar greenhouses. Comput. Electron. Agric..

[16] Ahamed M.S., Guo H., Tanino K.K. (2018). Sensitivity analysis of CSGHEAT model for estimation of heating consumption in a Chinese-style solar greenhouse. Comput. Electron. Agric..

[17] Tong G., Christopher D.M., Li B. (2009). Numerical modelling of temperature variations in a Chinese solar greenhouse. Comput. Electron. Agric..

[18] Sethi V.P. (2009). On the selection of shape and orientation of a greenhouse: Thermal modeling and experimental validation. Sol. Energy.

[19] Gavilan P., Ruiz N., Lozano D. (2015). Daily forecasting of reference and strawberry crop evapotranspiration in greenhouses in a Mediterranean climate based on solar radiation estimates. Agric. Water Manag..

[20] Coulson K.L. (1975). Solar and Terrestrial Radiation: Methods and Measurements.

[21] Inman R.H., Pedro H.T., Coimbra C.F. (2013). Solar forecasting methods for renewable energy integration. Prog. Energy Combust. Sci..

[22] Khorasanizadeh H., Mohammadi K., Goudarzi N. (2016). Prediction of horizontal diffuse solar radiation using clearness index based empirical models: A case study. Int. J. Hydrog. Energy.

[23] Muneer T., Hawas M.M., Sahili K. (1984). Correlation between hourly diffuse and global radiation for New Delhi. Energy Convers. Manag..

[24] Cabrera F.J., Baille A., Lopez J.C., Gonzalezreal M.M., Perezparra J. (2009). Effects of cover diffusive properties on the components of greenhouse solar radiation. Biosyst. Eng..

[25] Tong X., Sun Z., Sigrimis N., Li T. (2018). Energy sustainability performance of a sliding cover solar greenhouse: Solar energy capture aspects. Biosyst. Eng..

[26] Elmaghlany W.M. (2016). A novel analytical solution for the transmissivity of curved transparent surfaces with application to solar radiation. Appl. Therm. Eng..

[27] Baptista F.J., Bailey B.J., Meneses J.F. (2012). Effect of nocturnal ventilation on the occurrence of Botrytis cinerea in Mediterranean unheated tomato greenhouses. Crop Prot..

[28] Monteith J.L. (1965). Evaporation and Environment. Symp. Soc. Exp. Biol..

[29] Pereira L.S., Allen R.G., Smith M., Raes D. (2015). Crop evapotranspiration estimation with FAO56: Past and future. Agric. Water Manag..

[30] Hargreaves G.H., Samani Z. (1982). Estimating potential evapotranspiration. J. Irrig. Drain. Div..

[31] Yang Y., Cui Y., Bai K., Luo T., Dai J., Wang W., Luo Y. (2019). Short-term forecasting of daily reference evapotranspiration using the reduced-set Penman-Monteith model and public weather forecasts. Agric. Water Manag..

[32] Hargreaves G.H., Allen R.G. (2003). History and evaluation of hargreaves evapotranspiration equation. J. Irrig. Drain. Eng..

[33] Carmassi G., Incrocci L., Maggini R., Malorgio F., Tognoni F., Pardossi A. (2007). An aggregated model for water requirements of greenhouse tomato grown in closed rockwool culture with saline water. Agric. Water Manag..

[34] Troparevsky M.I., Dattellis C.E. (2004). On the convergence of the LMS algorithm in adaptive filtering. Signal Process..

[35] Parra I.E., Hernandez W., Fernandez E. (2013). On the convergence of LMS filters under periodic signals. Digit. Signal Process..

[36] Zhu Z., Gao X., Cao L., Pan D., Cai Y., Zhu Y. (2016). Analysis on the adaptive filter based on LMS algorithm. Optik.

[37] Tan L., Jiang J. (2019). Digital Signal Processing.

[38] Theodoridis S. (2015). Machine Learning.

[39] Lu W., Zhang Y., Fang H., Ke X., Yang Q. (2017). Modelling and experimental verification of the thermal performance of an active solar heat storage-release system in a Chinese solar greenhouse. Biosyst. Eng..

[40] Gutierrezcorea F.V., Mansocallejo M., Morenoregidor M.P., Velascogomez J. (2014). Spatial Estimation of Sub-Hour Global Horizontal Irradiance Based on Official Observations and Remote Sensors. Sensors.

[41] Bo Z., Yang J., Sun C., Jiang S. (2014). A filtered-x weighted accumulated LMS algorithm: Stochastic analysis and simulations for narrowband active noise control system. Signal Process..

[42] Nakamori S. (2013). Design of RLS-FIR filter using covariance information in linear continuous-time stochastic systems. Appl. Math. Comput..

[43] Korki M., Zayyani H. (2019). Weighted diffusion continuous mixed p-norm algorithm for distributed estimation in non-uniform noise environment. Signal Process..

[44] Singh T.S., Chatterjee A. (2011). A comparative study of adaptation algorithms for nonlinear system identification based on second order Volterra and bilinear polynomial filters. Measurement.

[45] Liu X., Wang Y., Geng J., Chen Z. (2013). Modeling of hysteresis in piezoelectric actuator based on adaptive filter. Sens. Actuators A Phys..

[46] Cooley J.W., Tukey J.W. (1965). An algorithm for the machine calculation of complex Fourier series. Math. Comput..

[47] Badescu V., Dumitrescu A. (2014). New types of simple non-linear models to compute solar global irradiance from cloud cover amount. J. Atmos. Sol. Terr. Phys..

[48] Badescu V., Dumitrescu A. (2013). New models to compute solar global hourly irradiation from point cloudiness. Energy Convers. Manag..

[49] Son J., Park Y., Lee J., Kim H. (2018). Sensorless PV power forecasting in grid-connected buildings through deep learning. Sensors.

[50] Gueymard C.A., Myers D.R., Badescu V. (2008). Validation and Ranking Methodologies for Solar Radiation Models. Modeling Solar Radiation at the Earth’s Surface: Recent Advances.

[51] Villarrubia G., De Paz J.F., La Iglesia D.H., Bajo J. (2017). Combining Multi-Agent Systems and Wireless Sensor Networks for Monitoring Crop Irrigation. Sensors.

