# Supplementary material for: Estimation of Solar Radiation for Tomato Water Requirement Calculation in Chinese-Style Solar Greenhouses Based on Least Mean Squares Filter
Source: Sensors (Basel). 2019 Dec 25;20(1):155. doi: 10.3390/s20010155 (PMC6983187; doi:10.3390/s20010155)
Supplement: Supplementary file 1 [file sensors-20-00155-s001.zip › sensors-659408-supplementary.docx]

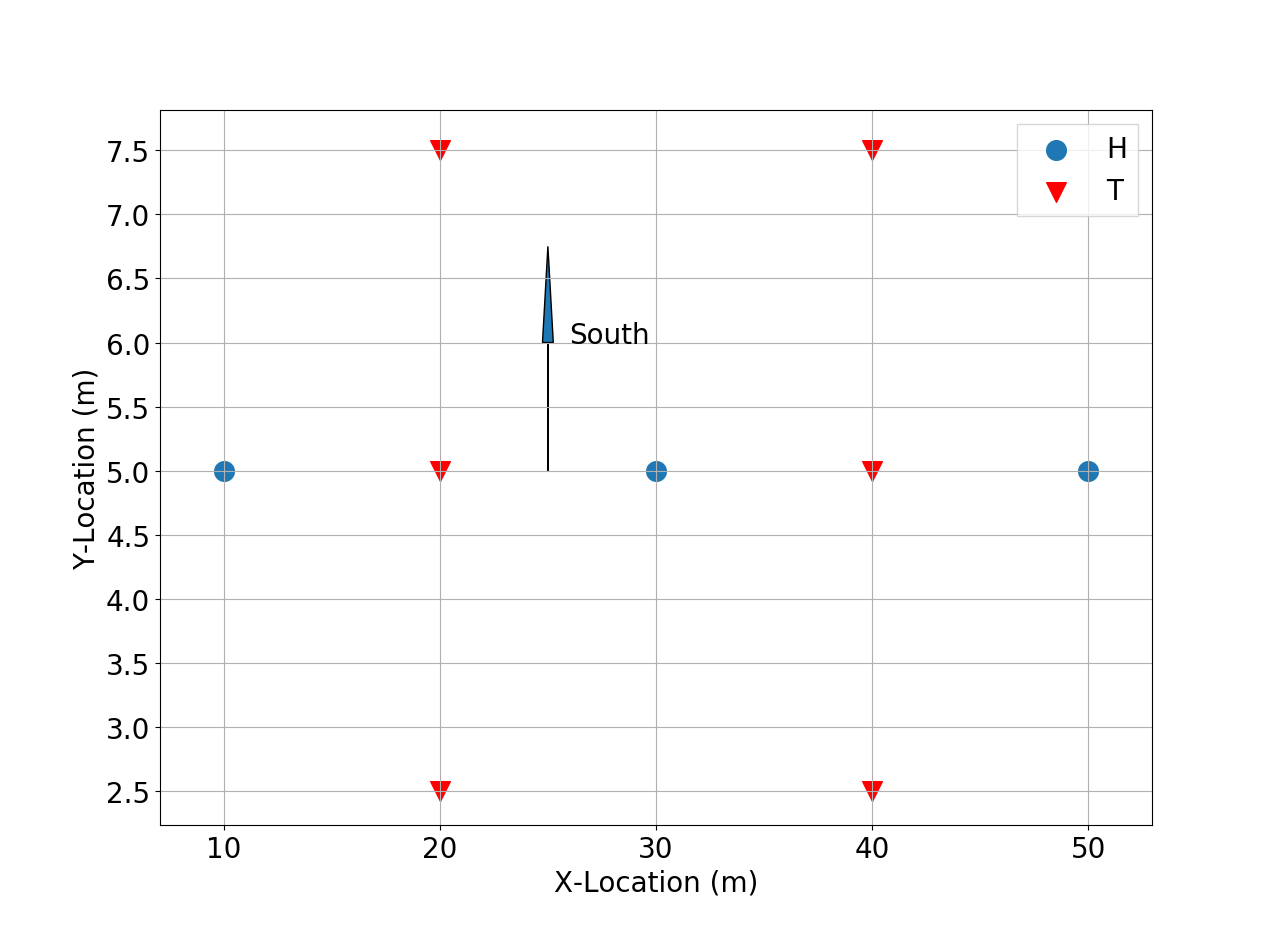


**Figure S1.** Diagram of sensor placement inside the greenhouse; H and T indicate the location of pyranometers and temperature sensors, respectively. The coordinate Y_Location = 0 is the north margin of cultivation area.


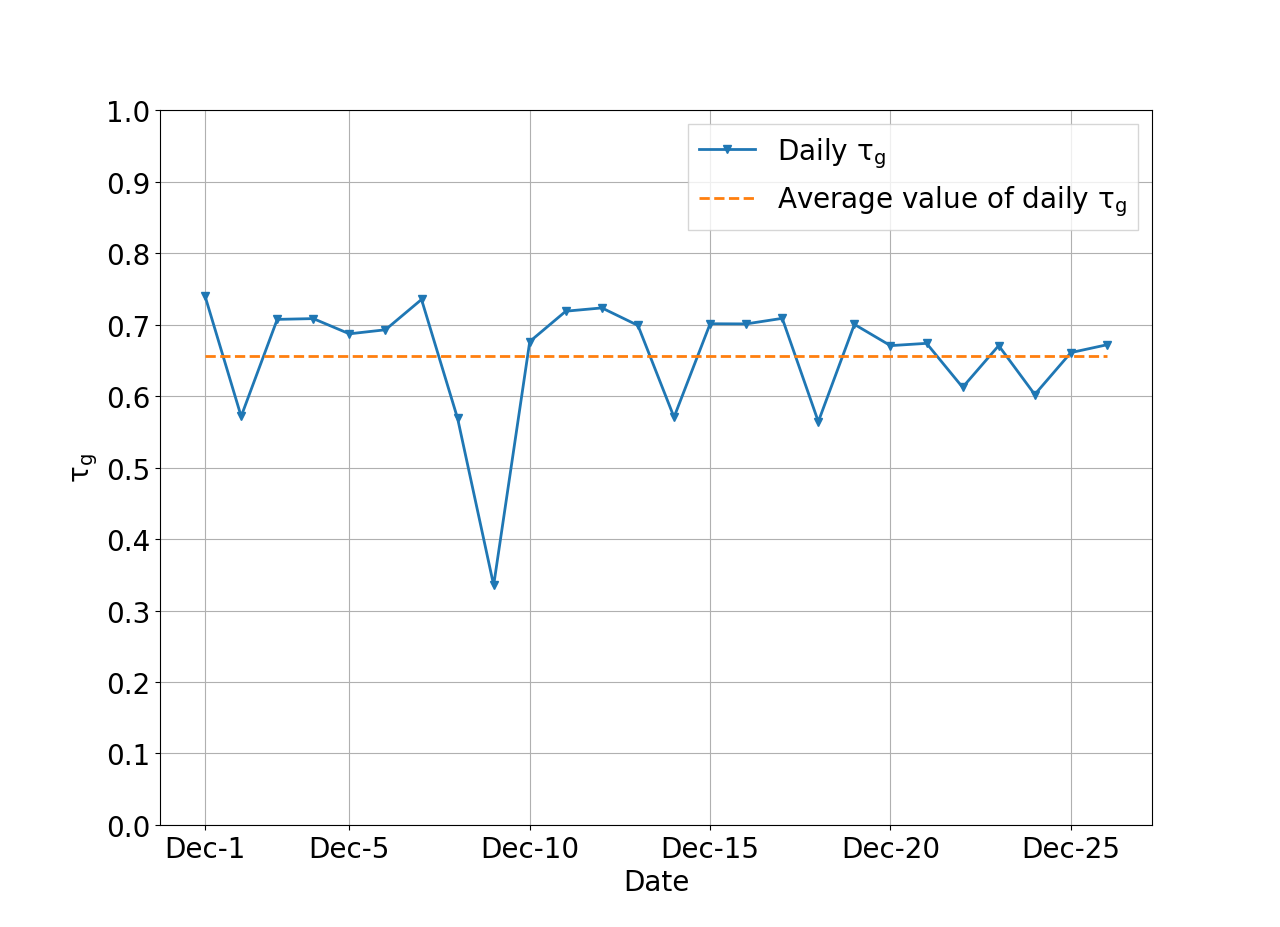


**Figure S2.** Daily average greenhouse global transmittance (τ_g_) calculated from data from exterior and interior inside sensors and the overall average value of τ_g_ during experiment.


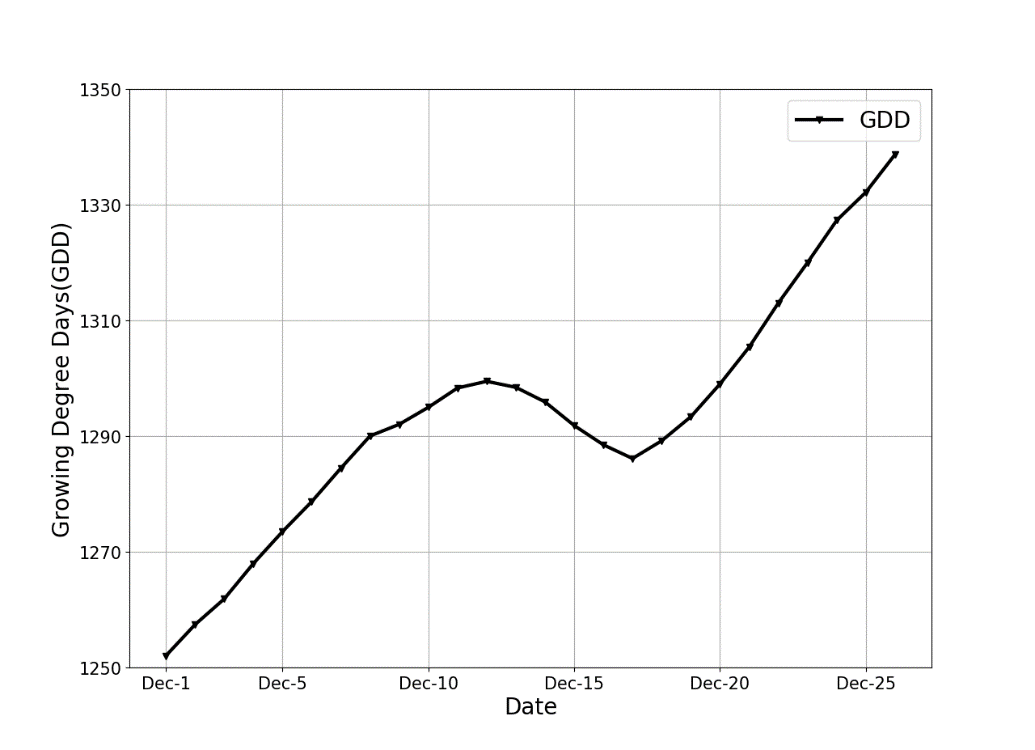


**Figure S3.** Growing degree days (GDD) during experimental days.

**Figure S4.** Basic diagram of adaptive filter, where y(n) is the output signal, x(n) is the input signal, d(n) is the desired output, and e(n), based on which the filter coefficients are refreshed, is the error signal of y(n) and d(n).
